# Supplementary material for: Phenotypic Variability of Root System Architecture Traits for Drought Tolerance among Accessions of Citron Watermelon (Citrullus lanatus var. citroides (L.H. Bailey)
Source: Plants (Basel). 2022 Sep 26;11(19):2522. doi: 10.3390/plants11192522 (PMC9573351; doi:10.3390/plants11192522)
Supplement: Supplementary file 1 [file plants-11-02522-s001.zip › plants-1868098-supplementary.pdf]

Supplementary Table S1: Linear equation showing growth rate of root traits parameters under non-stress condition

Accession	RSW	RSD	CHA	TRL	RBC	LA
WWM-09	*y*=0.285*x*+3.1936	*y*=1.0156*x*+2.8544	*y*=66.743*x*+ 134.28	*y*=2.1919*x*+7.7744	*y*=1.7459*x*+0.3995	*y*=2.5777x+6.4476
WWM-15	*y*=0.1911*x*+1.0574	*y*=0.8591*x*+1.2525	*y*=39.172*x*+140.2	*y*=1.6703*x*+2.1815	*y*=1.2634*x*-0.0688	*y*=1.9087x+2.8842
WWM-37(2)	*y*=0.2727*x*+3.0675	*y*=0.9091*x*+ 6.1064	*y*=58.729*x*+121.31	*y*=2.4986*x*+2.7289	*y*=1.2933*x*+1.1641	*y*=2.3793x+6.0579
WWM-39	*y*=0.2362*x*+2.4728	*y*=1.0472*x*+0.1188	*y*=48.044*x*-193.87	*y*=2.2071*x*-0.0919	*y*=1.4745*x*+2.4524	*y*=1.7311x+7.4163
WWM-41(A)	*y*=0.3383*x*+1.8819	*y*=1.0454*x*+0.3611	*y*=60.993*x*-270.18	*y*=2.2278x+6.3956	*y*=1.5074*x*+1.5953	*y*=1.9871x+10.389
WWM-46	*y*=0.1886*x*+1.6118	*y*=0.6928*x*+1.4568	*y*=29.129*x*-139.69	*y*=1.5573x+3.4118	*y*=1.0846*x*+2.8334	*y*=1.7452x+3.1535
WWM-64	*y*=0.252*x*+1.7947	*y*=0.9075*x*+2.3052	*y*=27.663*x*-55.776	*y*= 1.7825x+2.688	*y*=1.3216*x*+0.2302	*y*=1.9504x+3.6145
WWM-68	*y*=0.2798*x*+0.6244	*y*=0.8454*x*+1.0072	*y*=31.715*x*-120.66	*y*=1.5525x+4.7431	*y*=1.4527*x*-0.6349	*y*=1.2679x+4.9592
WWM-76	*y*=0.3855*x*+2.6358	*y*=1.1202*x*+1.658	*y*=71.403x-259.74	*y*=2.1392*x*+3.3597	*y*=1.832*x*+0.6747	*y*=1.7834*x*+5.2302

Supplementary Table S2: Linear equation showing growth rate of root traits parameters under water-stress condition

Accession	RSW	RSD	CHA	TRL	RBC	LA
WWM-09	*y*=0.3143x+3.5535	*y*=1.2778*x*+0.0309	*y*=78.593*x*-272.54	*y*=2.3251*x*+7.0479	*y*=1.800*x*+0.709	*y*=0.7621*x*+15.816
WWM-15	*y*=0.284*x*+0.7458	*y*=0.8887*x*-0.0488	*y*=40.728*x*-196.59	*y*=1.7794*x*+1.6983	*y*=1.2983*x*-1.5396	*y*=0.2784*x*+13.278
WWM-37(2)	*y*=0.3245*x*+2.8521	*y*=1.1015*x*+1.8807	*y*=68.341*x*-202.84	*y*=2.2065*x*+5.8417	*y*=1.4318*x*-0.844	*y*=0.7882*x*+16.941
WWM-39	*y*=0.2981*x*+2.294	*y*=1.1408*x*-1.443	*y*=60.276*x*-269.85	*y*=2.144*x*+1.8661	*y*=1.4823*x*+1.3281	*y*=0.669*x*+12.613
WWM-41(A)	*y*=0.4296*x*+1.5627	*y*=1.1517*x*-1.2085	*y*=79.814*x*-398.74	*y*=2.3619*x*+2.6562	*y*=1.6766*x*-1.1798	*y*=0.8964*x*+17.06
WWM-46	*y*=0.2956*x*+0.1546	*y*=0.8749*x*-0.5921	*y*=40.474*x*-230.57	*y*=1.7235*x*+0.8112	*y*=1.1967*x*+0.672	*y*=0.2769*x*+12.879
WWM-64	*y*=0.2207*x*+1.4792	*y*=0.8958*x*+0.7015	*y*=37.138*x*-153.22	*y*=1.7453*x*+3.1479	*y*=1.3168*x*-1.201	*y*=0.4031*x*+12.713
WWM-68	*y*=0.2921*x*+0.6008	*y*=0.877*x*+0.0255	*y*=41.477*x*-209.11	*y*=1.7787*x*+2.6726	*y*=1.3185*x*-1.4154	*y*=0.8038*x*+6.7596
WWM-76	*y*=0.403*x*+3.0356	*y*=1.2496*x*-1.0719	*y*=91.609*x*-414.62	*y*= 2.4631*x*+4.0502	*y*=1.6148*x*+0.9313	*y*=0.8939*x*+12.343

PAINT & HARDWARE

HAYFIELDS MICA HARDWARE
SHOP54, HAYFIELDS MALL
HAYFIELDS 3201
PH: 033-386 2464
VAT REG: 4130132808

Tax Invoice

Till 4 Sale # 223154 13:03:59 25/06/2021

Code / Description	Unit Price	Qty	Total
5240	15.99	1	15.99
TAPE PACKAGING BUFF 48MMX50M			
6933528730217	59.99	1	59.99
HACKSAW JNR 150MM TOLSEN			
HNNMN6	79.99	1	79.99
PATTEX NO MORE NAILS INVISIBLE 40G			

Vat Included	20.34
**Subtotal**	**155.97**
Sureswipe Online	155.97

Cashier KELLY-ANNE

Innovative Accounting Software by IQRetail

Thank you, please call again!
Retain slip for return and guarantee

Customer Details

PAINT & HARDWARE

HAYFIELDS MICA HARDWARE
SHOP54, HAYFIELDS MALL
HAYFIELDS 3201
PH: 033-386 2464
VAT REG: 4130132808

Tax Invoice

Till 3 Sale # 140500 11:26:09 03/07/2021

Code / Description	Unit Price	Qty	Total
6005633012660	10.99	1	10.99
CABLE TIES T50I 4.8x300MM BLK (10)			
6005633012660	10.99	1	10.99
CABLE TIES T50I 4.8x300MM BLK (10)			
6005633012660	10.99	1	10.99
CABLE TIES T50I 4.8x300MM BLK (10)			

Vat Included	4.30
**Subtotal**	**32.97**
Sureswipe Online	32.97

Cashier KELLY-ANNE

Innovative Accounting Software by IQRetail

Thank you, please call again!
Retain slip for return and guarantee

Customer Details

PAINT & HARDWARE

HAYFIELDS MICA HARDWARE
KwaZulu Natal
033 386 2464

25/06/2021 13:03:57

PURCHASE TILL : 4

CUSTOMER COPY

MERCHANT ID : 360000000204630
TERMINAL ID : 89000746
RETRIEVAL NUMBER: 400060004454
AUTHORIZATION: 002734
CARD NUMBER: ****************9179
CARD HOLDER :
EMV AID : A0000000031010
EMV TVR : 0000000000
EMV APP LABEL:GOLD CHEQUE CARD
UUID:203878a5-36a4-4475-ab57-2895fde0c71d

STATUS : 00-Approved

TOTAL
ZAR155.97

THANK YOU

Sureswipe Integrated Payments

PAINT & HARDWARE

HAYFIELDS MICA HARDWARE
KwaZulu Natal
033 386 2464

03/07/2021 11:26:06

PURCHASE TILL : 3

CUSTOMER COPY

MERCHANT ID : 3600000002[illegible]1630
TERMINAL ID : 89000747
RETRIEVAL NUMBER: 401000003091
AUTHORIZATION: 331260
CARD NUMBER: ****************97[illegible]
CARD HOLDER : /
EMV AID : A0000000031010
EMV TVR : 0000000000
EMV APP LABEL:VISA GOLD
UUID:220f52d2-c13[illegible]-4c5f-9504-062c4871571b

STATUS : 00-Approved

TOTAL ZAR32.97

THANK YOU

Sureswipe Integrated Payments

FICE
Street, Pretoria West
02, Pretoria West 0117
PROPRIETARY LIMITED Reg No. 1969/017998/07

Tel: (012) 352 2000
Fax: (012) 327 1994 (Sales)
Fax: (012) 327 1997 (Admin)
VAT Reg. No. 4750111470

SIGNAGE MATERIALS & SUPPLIES
maizey
ENGINEERING PLASTIC PRODUCTS

ounts: admin@maizey.co.za • General Enquiries: nat.sales@maizey.co.za • NATIONAL NUMBER: 086 1100 420 • WEBSITE: www.maizey.co.za

A L
ON

BELLVILLE
BLOEMFONTEIN
CAPE TOWN
DURBAN
EAST LONDON
EDENVALE
GEORGE
GERMISTON
JOHANNESBURG
KLERKSDORP
NELSPRUIT
PIETERMARITZBURG
PINETOWN
POLOKWANE
PORT ELIZABETH
PRETORIA EAST
PRETORIA WEST
RICHARDS BAY
ROODEPOORT
RUSTENBURG
VEREENIGING
WELKOM
WITBANK
ENGINEERING PLASTICS

Bank Details: Account Name: **Maizey's (Pty) Ltd** Bank: **FNB** Account No.: **51070040443** Branch Code: **261-550** Branch: **Centurion**

DELIVER TO

CUSTOMER VAT NUMBER

COPY TAX INVOICE
DELIVERY NOTE

Formatt ☎ 010 900 4496 (MAI 0002 001)

NT NUMBER	OUR ORDER NUMBER	YOUR ORDER NUMBER	TERMS	PAGE NO.	DATE	BRANCH

CT CODE	DESCRIPTION	UNIT	QUANTITY	UNIT PRICE	GROSS AMOUNT	DISC. %	NETT AMOUNT

NOTES

SUB TOTAL	
VAT	
**TOTAL**	

knowledges the order of above goods and services and accepts the STANDARD CONDITIONS OF AGREEMENT on the reverse side. The
wledges that the above amount is due unconditionally and that it is payable
ery, or
er is an **Approved Customer**,
from the date of an Invoice being issued by the Company.
heque: The Customer hereby declares that there is sufficient funds available and that such funds will remain available in order that all cheque
honoured; and that under **no circumstances** will any cheque be stopped.

Follow us on social media or shop our products online via the Maizey Shop!

1/21 NAME (PRINT) TAKUDZWA MANDIZVO SIGNATURE

CUSTOMER COPY
